# Neurobehavioural challenges experienced by HIV exposed infants: a study in South Africa

**DOI:** 10.1186/s12887-022-03526-5

**Published:** 2022-08-06

**Authors:** Gina Rencken, Pragashnie Govender, Catharina J. E. Uys

**Affiliations:** 1 Department of Occupational Therapy, University of Kwazulu Natal (South Africa), Durban, South Africa; 2 Department of Occupational Therapy, University of KwaZulu-Natal, Durban, South Africa; 3 Department of Occupational Therapy, University of Pretoria (South Africa), HW Snyman South Building 5-19, Prinshof Campus, ), Durban, South Africa

**Keywords:** HIV, EPDS, Maternal depression, NBAS, Neurobehavioural functioning

## Abstract

**Background:**

The newborn infant is a complexly organized, competent being, who plays an active role in shaping their environment through their increasing skills in autonomic regulation, motor control, regulation of state and social interaction. Infants born to HIV positive mothers, are exposed to HIV and antiretroviral therapy inutero, and may experience adverse effects from this.

**Methods:**

A cross-sectional study of 132 mother-infant dyads from a large public health hospital in South Africa. Infants were assessed using the Neonatal Behavioural Assessment Scale on day two of life, and mothers mental health assessed using the Edinburugh Postnatal Depression Scale. Medical and demographic data on mothers and infants was collected, including maternal age, HIV status, length of time on antiretrovirals, relationship status, employment status, gravid status, mode of delivery, infant anthropometrics and infant gender. Data was input into IBM SPSS statistics 21, where frequencies and percentages for descriptive analysis, and Chi-square and student’s two sample t-tests were run to compare data from HIV infected-exposed and HIV uninfected-unexposed mothers and infants.

**Results:**

HIV exposed infants were smaller than HIV unexposed infants, even though low birth weight was an exclusion criteria. Statistically significant differences were found between HIV exposed and unexposed infants in neurobehavioiral items of social interaction (*p* = 0.00), motor system (*p* = 0.00) and state organization (*p* = 0.01), with HIV exposed infants performing less optimally in these domains. HIV exposed infants also presented with more abnormal reflexes. Infants born to depressed mothers showed superior motor skills, state organization and state regulation than infants born to mothers who did not score in the possibly depressed range.

**Conclusions:**

HIV exposed infants have inferior neurobehavioural functioning, which may affect their quality of life and ability to develop a reciprocal relationship with a primary caregiver. This may have an effect on development, behaviour and mental health in later childhood. HIV exposed infants shoud be monitored closely and their functioning in autonomic stability, motor control, resualtion of state and social interaction assessed regularly. Guidance for caregivers in incorporating strategies into the care of these infants is essential to buffer the possible long term negative effects on development.

## Background

The newborn infant is a complexly organized, competent being who plays an active role in shaping their development by communicating with their caregiver and drawing out the specific caregiving and support they need to adapt to the extra-uterine environment [1]*.* Newborn infants have a specific developmental agenda after birth. They acquire skills in four neurobehavioral domains, namely, regulating their autonomic nervous system, regulating their behavioral state, controlling their motor behavior and interaction with animate and inanimate objects in their immediate environment. Assessing an infant’s neurobehavioral functioning in the four domains provides a thorough portrayal of their competencies and detect areas where support may be required, guiding responsive caregiving [1].

Newborn behavior is dependent on genetic factors and several variables influencing the intrauterine environment. The Neurobehavioral Assessment Scale (NBAS) is a comprehensive tool to evaluate infant neurobehaviour in the first two months of life across the four domains, as the infant adapts to the world and develops competency. It allows for variability in newborn infant behavior and prenatal influences. Various factors from the mother, infant, labour and childbirth, social environment, and demographics influence neonatal behavior in the perinatal period [1, 2]. Maternal factors include stress, mental health, drug exposure and health problems [1, 3–5]. Drug exposure and health problems can include HIV infection and prescribed medication, such as antiretroviral therapy (ART) taken by the mother to prevent transmission to her infant.

South Africa is estimated to have more than 7,5 million people living with HIV, with new infections surpassing 240 000 yearly between 2010 and 2019 [6–8]. With the adoption of the World Health Organization (WHO) treatment guidelines option B + , in which HIV infected pregnant women receive ART regardless of disease staging or CD4 count, the prevalence of mother to child transmission (MTCT) has decreased from near 40% to lower than 2% [9, 10].

Infants born to mothers who are infected with HIV are exposed to the virus in-utero, and the various ARTs that the mother consumes through HIV-specific pathways. These pathways include direct exposure to the neurotropic HIV-virus, the effects of maternal inflammation and immune activation in-utero, immune activation and inflammation in the exposed fetus, which all influence brain development, and ART toxicity [11]. Evidence from multiple studies indicates the adverse effects experienced by these infants in their early developmental years [12–16]. These adverse effects include mitochondrial dysfunction, most often observed in laboratory abnormalities seizures, cognitive and motor delays, cardiac dysfunction, and slower earlier growth [12, 14]. In assessing neuropsychological development, HIV exposed infants had lower neurodevelopmental outcomes than their unexposed counterparts, even when controlling for confounders such as poverty, nutritional status, and maternal education, which are known to negatively influence these outcomes [12]. HIV exposed infants assessed with the Bayley Scales of development at 25 months showed inferior receptive language, social-emotional and self-regulatory skills [17]. There are limited studies that have assessed infant behavior in the early weeks. The NBAS indicated that HIV exposed infants had poorer orientation and reflex scores than infants born to seronegative mothers. However, there is no information about ART use in their mothers [18]. HIV exposed infants also have more feeding difficulties, specifically with regurgitating food, slowed feeding and vomiting [19]. Some ART can cause neuropsychiatric effects like depression, emotional stress and anxiety among HIV positive pregnant women [10, 12]. Manikkam and Burns (2012) identified HIV seropositivity as a significant risk factor for depression among pregnant women in South Africa [20].

Infants born to depressed mothers are compromised at birth and demonstrate dysregulation over the first month of life [21, 22]. These infants presented with difficulties in the state regulation and social interaction domains, according to NBAS scores. They were more aroused and less attentive than infants born to non-depressed mothers. These infants received lower scores on orientation to live face and voice stimuli, alertness items, cuddliness and hand to mouth actions and are described as more irritable, less consolable and more excitable [21, 23–26].

The WHO guidelines for improving early childhood development focus on nurturing care. This concept includes promoting health and optimal nutrition, a stable environment, and opportunities for early learning through affectionate interactions and relationships [27]. There is a specific focus on the period from pregnancy to the age of three, where critical periods for receiving nurturing care are present, and the fetus and infant are most sensitive to intervention and most susceptible to adverse events [28]. The WHO declared that as an essential measure for sustainable development, enabling each young child to reach their full developmental potential is a human right, starting in pregnancy and early infancy [27].

There is a notable lack of studies on the HIV exposed infant’s neurodevelopment and behavior in early infancy within the need for early monitoring in the changing landscape of HIV prevention.

## Methods

### Aim, study design, and setting

This study aimed to explore the neurobehavioral functioning of HIV exposed infants born to mothers seropositive to HIV-1. The objectives were to observe the neurobehavioral functioning of HIV exposed infants assessed on the NBAS [1] and compare these to the neurobehavioral functioning of unexposed infants. It further aimed to correlate infant neurobehavior to self-reported maternal mental health measured on the Edinburgh Postnatal Depression Scale (EPDS) [29]. An exploratory cross-sectional cohort study was completed at a large academic hospital in KwaZulu-Natal (KZN), South Africa, over a six-month period [30].

### Participants and process

A preliminary visit was done to the obstetric ward prior to the commencement of the study in order to determine the population size. The ward intake book, which details maternal age, mode of delivery and adverse events (such as foetal distress, meconium-stained liquor, stillbirth, NICU admission or maternal high care transfer), infant gender, and a singleton or multiple birth, was perused for the number of admissions which would meet the maternal and infant inclusion criteria. These criteria were: the mothers had to be of adult maternal age (21 years – 39 years) residing in the EThekwini municipality of KZN and booked for delivery at the academic hospital. They needed to have attended at least two antenatal appointments, a singleton pregnancy, a completed HIV test and results available, and received ART in option B + if HIV positive. The infant had to be a well-baby (indicating that they only need standard practice neonatal nursing or medical care), attain APGAR scores at or above 7/10, be in the care of their mother in the obstetric ward, delivered at or after 38 weeks gestation. A total of 130 admissions meeting these criteria were noted in a month. There were no available published studies in this population to assist in calculating the sample size with a power analysis, and the researcher aimed for a sample size of 50 in each of the two mother-infant dyad groups (HIV positive mothers with exposed infants and HIV negative mothers with unexposed infants) in line with previous studies [18] and statistical guidance. Considering the fact that the only previous study on a similar population was published in 1997, prior to the widespread use of ART during pregnancy to prevent vertical transmission of HIV-1 [18], an exploratory study in the present-day South African context was warranted.

The study sample comprised 132 mother-infant dyads. After screening the ward intake log, mothers meeting the inclusion criteria were invited and recruited into the study with their infant, following additional screening through a discussion about illnesses or complications experienced during their pregnancy (such as diabetes mellitus, alcohol use, smoking, drug use, significant infections or pre-eclampsia) or with their infant directly after birth, and gaining informed consent. The hospital used in the research is a baby-friendly hospital, which strongly encourages breastfeeding and attachment. Infants are placed on their mother’s chest after delivery and sleep in the hospital bed with their mother, with the only period of separation being when the mother goes to do her ablutions, shower, or collect food from the trolley in the ward. Infants needing specialized care are transferred to the high care or intensive care units, and these infants were excluded from the study. The researcher completed the NBAS prior to reviewing the maternal or infant medical records in order to maintain blindness to HIV status prior to testing the infant. A total of 80 HIV-positive mothers and their exposed infants and 52 HIV-negative mothers and their unexposed infants participated in the study at the initial point of contact in the obstetric ward.

### Measures

Infants were assessed using the NBAS [1] in the presence of their mother on day two of life before being discharged to a step-down facility or home. The NBAS is a comprehensive neurobehavioral assessment used worldwide in research and clinical settings and is sensitive to subtle environmental effects and pre-, peri, and postnatal variables [1]. It is based on the assumption that the neonate is competent and complexly organized. The infant’s behavior was assessed on 28 behavioral items and 20 reflex items. The behavioral items (habituation, orientation, motor, range of state, regulation of state, autonomic stability) are scored on a nine-point likert scale. In contrast, the reflex items are scored on a four-point scale. The researcher is an experienced clinician in Occupational Therapy, with appropriate training in the administration and interpretation of the NBAS assessment. As an additional measure of reliability, the researcher watched the NBAS administration training video at the beginning of each week of data collection to ensure administration remained accurate. A sample of 20 assessment forms with in-depth descriptions of the infant’s behaviour in each of the 28 behavioral and 20 elicited items were independently scored and later compared to the scores given by the researcher. These compared scores were manually computed, and reached a 99% match on the behavioural items, and a 100% match on the elicited items, meeting inter-rater reliability criteria of the NBAS [1].

The infant’s medical file was perused after the NBAS assessment had been completed to address potential examiner bias. The medical files provided birth type, APGAR scores, weight, length, head circumference, HIV exposure, HIV-prophylactic medication if HIV exposed [31] and immunizations received. The method of feeding was verified with the infant’s mother.

Maternal medical data were accessed from the medical file after examination of the infant. Information gathered included HIV status, the length of time the mother had been taking ART if she is HIV positive [31], and age. Demographic information such as relationship status and employment status was gained from the mother. In addition, the mother independently completed the EPDS to assess her mental health whilst the researcher was in the ward and available to answer questions for clarity. The EPDS is a 10 item self-report questionnaire assessing the symptoms of depression. It focuses on cognitive and affective symptoms, omitting somatic symptoms often associated with depression (headaches, nausea, change in weight, and appetite), which may be confounded by pregnancy, and is thus a reliable screening tool for “perinatal” depression. Each reported item is scored on a four-point likert scale, with a total range of 0–30. It assesses how the responder has been feeling in the last seven days. A cut-off score of ≥ 10 is considered indicative of possible depression [29]. The scale has been validated in a previous South African study [20].

### Ethical considerations

Ethical approval was granted by the KZN Health Research and Knowledge Management Directorate of the provincial Department of Health (ref no. HRKM320/15 K2_2015RP40_914) and the Biomedical Research Ethics Committee of the University of KwaZulu-Natal (approval number BFC354/15). Permission was sought and granted by the Hospital CEO, the Matron in charge of Obstetric Nursing, and the sister in charge of the ward to access the unit. All mothers read the participant information sheet, read and signed informed consent independently prior to testing. There was no change to routine nursing or medical care as a result of participation or non-participation in the study.

### Statistical analysis

Data from the NBAS, EPDS, and medical files were input to Microsoft excel 2016 and imported into IBM SPSS Statistics 21. Frequencies and percentages were used to describe categorical data while continuous data were summarized in means and standard deviations (SD), with a 95% confidence interval. Demographic variables were analyzed using Chi-square (χ2) tests and student’s two-sample t-tests, with equal variations assumed or not assumed depending on Levine’s test for equality of variances. NBAS behavioral and reflex items were analyzed in the seven-cluster scoring system developed by Lester and colleagues. This scoring system is widely used in research and allows for effective data reduction by reducing the behavioral items into seven clusters. These include habituation (4 items), orientation (7 items), motor (5 items), range of state (4 items), regulation of state (4 items), autonomic stability (3 items), and reflexes (20 items) [1]. T-tests were computed to compare the data collected from the HIV exposed and unexposed infants and their mothers. Missing data occurred in the NBAS assessments of some infants: in the habituation package if they were not asleep at the time of the assessment, or woke up during the administration of the items, and if the assessment had to be stopped because the infant became too dysregulated or was too fragile to complete [1]. In the cases of these infants, scores were calculated for the items they were able to participate in.

## Results

A total of 132 mother-infant pairs participated in the study. Mothers had a mean age of 29 years (median 29.5 years, range 17 years). A total of 60.6% (80) were seropositive to HIV-1 and had been on ARTs for an average of 3.53 years. EPDS scores in the maternal population indicated 72% (95) of mothers reporting depression, with 59%(56) of these mothers being HIV positive, and 41% (39) being HIV negative. There was no statistical significance found between the prevalence of depression in the two groups in an independent samples t-test with equal variances assumed (*p* = 0.753). There were 79 (59.8%)male and 53 (40.2%)female infants. Caesarian section deliveries under a spinal block were performed for mothers of 60.6% (80) of these infants. Exclusive breastfeeding was followed by 118 (89.4%) of the mother-infant pairs. There was a significant correlation between infant anthropometrics and HIV exposure, with HIV exposed infants being smaller than non-exposed infants, even when low birth weight (LBW) (under 2 kg) was an exclusion criterion.

Table 1 indicates the means and standard deviations of maternal and infant characteristics, with correlations between HIV-positive mothers with their exposed infants and HIV-negative mothers and their unexposed infants. There were statistically significant differences between exposed and unexposed infants in the total section scores for the behavioral items of social interaction (*p* < 0.01), motor system (*p* < 0.01) and state organization (*p* = 0.01), as well as a number of abnormal reflexes present (*p* < 0.01), with the HIV exposed infants receiving inferior scores.

**Table 1:** NBAS section mean scores with maternal and infant population characteristics

	**HIV positive/exposed** **Mean(SD)** ***n* = 80**	**HIV negative/unexposed** **Mean(SD)** ***n* = 52**	**two-sample t-test** **Significance** ***p* ≤ 0.05**
**Maternal characteristics**			
Maternal age	29.074.91)	27.79(4.71)	*p* = 0.01*^#^
EPDS score	12.19(5.50)	13.04(4.69)	*p* = 0.35^#^
**Infant characteristics**			
Infant birthweight	3.24(0.41)	3.49(0.38)	*p* < 0.01*^#^
Infant head circumference	34.80(1.39)	35.92(1.22)	*p* < 0.01*^#^
Infant length	48.59(3.49)	51.29(2.98)	*p* < 0.01*^#^
**NBAS section scores**			
Habituation	*n* = 63 4.92(2.06)	*n* = 38 5.41(2.27)	*p* = 0.27^#^
Social interaction	*n* = 78 2.62(1.38)	*n* = 52 3.44(1.73)	*p* < 0.01*^#^
Motor system	4.21(0.79)	4.71(0.87)	*p* < 0.01*^#^
State organization	3.50(0.51)	3.82(0.66)	*p* = 0.01*^@^
State regulation	4.20(1.53)	4.63(1.77)	*p* = 0.14^#^
Autonomic system	5.49(1.50)	5.40(1.34)	*p* = 0.73^#^
Total abnormal reflexes	5.69(2.74)	2.06(1.75)	*p* < 0.01*^@^

^#^Equal variance assumed according to Levine’s test for equality of variances

^@^Equal variances not assumed according to Levine’s test for equality of variances

^*^p value significant at the 95% confidence level

A closer analysis of the 52 items (27 behavioral items, seven supplementary and 18 reflex items) measured shows a statistically significant difference in most scores achieved in social interaction, motor system, supplementary items and reflexes, with HIV exposed infants receiving inferior scores. Half of the items in the habituation and state organization sections showed inferior performance of HIV exposed infants (Table 2). Not all infants were in the appropriate sleep state at the beginning of the assessment to assess the habituation items. Some woke up during the administration of the response decrement (habituation) items. The examination had to be abandoned for some infants before all the reflex items were administered, as the cost of attention was too high, the infant became significantly dysregulated or became inconsolable.

**Table 2:** NBAS results in all items

Item	HIV exposure Mean(SD)		two sample t-test significance
	**Exposed (*n* = 80)**	**Unexposed (*n* = 52)**	**Significance** ***p* ≤ 0.05**
**BEHAVIOURAL ITEMS**			
** HABITUATION** ** Administered in states 1 to 3. Evaluation of response decrement to external stimulation of light, sound and touch. A measure of how well an infant protects their sleep**			
** Response dec.to light**	*n* = 63 4.92(2.29)	*n* = 38 4.92(2.52)	*p* = 1.00
** Response dec.to rattle**	*n* = 59 4.98(2.52)	*n* = 32 6.38(2.18)	*p* = 0.01*
** Response dec.to bell**	*n* = 49 5.14(2.81)	*n* = 29 7.00(2.05)	*p* < 0.01*
** Response dec.to foot probe**	*n* = 45 5.56(2.06)	*n* = 28 6.29(1.86)	*p* = 0.13
*Note: Where n value is less than 80 in the exposed infant group, and less than 52 in the unexposed infant group, it is due to the infant being in an awake state when the assessment commenced or waking up during administration of the habituation package*			
** SOCIAL INTERACTIVE** ** Administered in a quiet alert state (state 4).Evaluation of the infant’s orientation to and interaction with animate (person) and inanimate (rattle and ball) visual and auditory stimuli**			
** Animate Visual**	*n* = 78 2.53(2.00)	3.00(1.89)	*p* = 0.18
** Animate Visual & auditory**	*n* = 78 2.69(2.01)	3.42(2.02)	*p* = 0.05*^#^
** Inanimate visual**	*n* = 78 2.03(1.71)	3.02(2.04)	*p* < 0.01*^#^
** Inanimate visual & auditory**	*n* = 78 2.41(1.59)	2.77(2.03)	*p* = 0.26^#^
** Inanimate auditory**	*n* = 78 2.59(1.36)	3.67(2.05)	*p* < 0.01*^@^
** Animate auditory**	*n* = 78 3.28(1.76)	4.02(2.16)	*p* = 0.03*^#^
** Alertness**	*n* = 78 2.81(1.30)	4.21(1.94)	*p* < 0.01*^@^
*Note: Where n value is less than 80 in the exposed infant group, and less than 52 in the unexposed infant group, it is due to the infant not being able to reach and maintain a state of arousal where the social interactive package could be administered. This may be due to the infant being too sleepy, crying and needing consoling measures, or being too fragile to complete the assessment*			
** MOTOR SYSTEM** ** Assessed in alert states (3 to 5). Evaluation of the motor responses of the infant to handling**			
** General tone**	4.94(1.18)	5.50(1.06)	*p* = 0.01*^#^
** Motor maturity**	4.60(1.80)	4.83(1.41)	*p* = 0.42^@^
** Pull-to-sit**	4.03(1.04)	4.67(0.68)	*p* < 0.01*^@^
** Defensive**	4.00(1.90)	4.12(2.25)	*p* = 0.76^@^
** Activity level**	3.48(1.03)	4.21(1.00)	*p* < 0.01*^#^
** STATE ORGANI***Z***ATION** ** Assessed throughout the evaluation. A measure of the amount of motor and crying activity observed, as well as how the infant moves between states 1 to 6**			
** Peak of excitement**	2.54(0.99)	3.35(1.15)	*p* < 0.01*^@^
** Rapidity of build-up**	5.70(2.67)	5.00(2.76)	*p* = 0.15^#^
** Irritability**	2.78(1.82)	3.42(1.95)	*p* = 0.05*^#^
** Lability of states**	2.94(1.13)	3.33(1.06)	*p* = 0.05*^#^
** STATE REGULATION** ** Assessed throughout the evaluation. A measure of the ability of the infant to move between and maintain their state of arousal in alert or sleep states, and the strategies used or needed to facilitate this**			
** Cuddliness**	6.51(1.56)	6.75(1.28)	*p* = 0.36^#^
** Consolability**	3.19(2.16)	*n* = 38 3.61(2.02)	*p* = 0.34^#^
** Self-quieting**	3.47(2.79)	4.45(2.65)	*p* = 0.07^#^
** Hand-to-mouth**	2.99(2.73)	3.48(2.73)	*p* = 0.31^#^
** AUTONOMIC SYSTEM** ** Assessed throughout the evaluation by observing the changes in the infant’s sk**in colour and vascularity, and in observations of tremors and startles-giving indication of central nervous system functioning and how the central and autonomic nervous systems are able to adapt to changes in the environment			
** Tremulousness**	5.91(1.78)	5.44(2.31)	*p* = 0.26^#^
** Startles**	6.81(1.66)	6.58(1.99)	*p* = 0.46^#^
** Lability of skin colour**	3.40(1.22)	4.23(1.08)	*p* < 0.01*^#^
** SUPPLEMENTARY ITEMS** ** Assessed throughout the evaluation. A measure of the qualitative aspects of the infant’s performance**			
** Quality of alertness**	2.45(1.01)	3.42(1.72)	*p* < 0.01*^@^
** Cost of attention**	4.21(1.47)	5.23(1.94)	*p* < 0.01*^@^
** Examiner facilitation**	3.76(1.63)	4.85(1.93)	*p* < 0.01*^@^
** General irritability**	3.85(2.59)	5.29(2.80)	*p* < 0.01*^#^
** Robustness & Endurance**	3.50(1.26)	5.23(2.10)	*p* < 0.01*^@^
** State regulation**	3.48(1.51)	5.04(2.01)	*p* < 0.01*^#^
** Examiners emotional response**	4.19(2.03)	6.15(2.40)	*p* < 0.01*^@^
** REFLEXES** ** Administered in an alert state 3 – 5. Evaluation of reflex items with infant in supine with head in midline**			
** Plantar**	1.73(0.50)	1.98(0.24)	*p* < 0.01*^@^
** Babinski**	1.74(0.59)	2.04(0.39)	*p* < 0.01*^@^
** Ankle clonus**	0.46(0.62)	0.56(0.57)	*p* = 0.37^#^
** Rooting**	1.73(0.52)	1.98(0.24)	*p* < 0.01*^@^
** Sucking**	1.81(0.48)	2.04(0.19)	*p* < 0.01*^@^
** Glabella**	2.03(0.45)	2.02(0.46)	*p* = 0.94^#^
** Passive resistance – legs**	*n* = 78 1.68(0.76)	2.08(0.436)	*p* < 0.01*^@^
** Passive resistance – arms**	*n* = 78 1.87(0.65)	2.12(0.40)	*p* = 0.01*^@^
** Palmar**	*n* = 79 1.56(0.50)	1.90(0.30)	*p* < 0.01*^@^
** Placing**	*n* = 77 1.71(0.46)	1.81(0.40)	*p* = 0.22^@^
** Standing**	*n* = 77 1.51(0.50)	1.65(0.56)	*p* = 0.12^#^
** Walking**	*n* = 77 1.57(0.76)	1.67(0.65)	*p* = 0.44^#^
** Crawling**	*n* = 77 1.57(0.68)	1.75(0.44)	*p* = 0.07^@^
** Incurvation**	*n* = 77 1.51(0.87)	1.87(0.35)	*p* < 0.01*^@^
** Tonic deviation head& eyes**	*n* = 77 1.75(0.46)	*n* = 51 2.00(0.00)	*p* < 0.01*^@^
** Nystagmus**	*n* = 59 0.85(0.64)	*n* = 42 1.12(0.45)	*p* = 0.01*^@^
** TNR**	*n* = 77 1.34(0.55)	*n* = 51 1.61(0.49)	*p* = 0.01*^#^
** Moro**	*n* = 77 1.73(0.50)	1.96(0.34)	*p* < 0.01*^@^
*Note: Where n value is less than 80 in the exposed infant group, and less than 52 in the unexposed infant group, it is due to the infant not being able to complete the assessment. This may be due to the infant being too sleepy, crying and needing consoling measures, or being too fragile to complete the assessment*			

^#^Equal variance assumed according to Levine’s test for equality of variances

^@^Equal variances not assumed according to Levine’s test for equality of variances

^*^p value significant at the 95% confidence level

Significant differences between the behavioural states of infants born to mothers with possible depression and those born to mothers who do not report depression were noted. The infants born to depressed mothers showed superior motor skills, state organization and state regulation (Table 3).

**Table 3:** Maternal mental health and HIV status correlations with NBAS section scores

	**Possibly depressed**		**Not possibly depressed**		**independent two-sample t-test significance**	
	**Mean(SD)** ***n* = 97**		**Mean(SD)** ***n* = 35**		**Significance** ***p* ≤ 0.05**	
**Habituation**	*n* = 72 5.18(2.04)		*n* = 29 4.90(2.39)		0.55^#^	
	**HIV + **	**HIV-**	**HIV + **	**HIV-**	**HIV + **	**HIV-**
	*n* = 41 4.74(1,97)	*n* = 31 5.77(2.02)	*n* = 22 5.25(2.22)	*n* = 7 3.80)2.76)	0.36^#^	0.04*^#^
**Social interaction**	*n* = 96 3.00(1.57)		*n* = 34 2.81(1.62)		0.57^#^	
	**HIV + **	**HIV-**	**HIV + **	**HIV-**	**HIV + **	**HIV-**
	*n* = 57 2.61(1.21)	*n* = 39 3.57(1.85)	*n* = 21 2.67(1.80)	*n* = 13 3.51(1.30)	0.89^@^	0.36^#^
**Motor system**	*n* = 97 4.50(0.80)		*n* = 35 4.13(0.96)		0.03*^#^	
	**HIV + **	**HIV-**	**HIV + **	**HIV-**	**HIV + **	**HIV-**
	*n* = 58 4.23(0.72)	*n* = 39 4.90(0.76)	*n* = 22 4.13(0.98)	*n* = 13 4.14(0.96)	0.61^#^	0.01*^#^
**State organization**	*n* = 97 3.69(0.56)		*n* = 35 3.46(0.64)		0.05*^#^	
	**HIV + **	**HIV-**	**HIV + **	**HIV-**	**HIV + **	**HIV-**
	*n* = 58 3.55(0.46)	*n* = 39 3.90(0.63)	*n* = 22 3.40(0.61)	*n* = 13 3.58(0.70)	0.24^#^	0.13^#^
**State regulation**	*n* = 97 4.52(1.75)		*n* = 35 3.96(1.17)		0.04*^@^	
	**HIV + **	**HIV-**	**HIV + **	**HIV-**	**HIV + **	**HIV-**
	*n* = 58 4.28(1.64)	*n* = 39 4.89(1.87)	*n* = 22 4.01(1.19)	*n* = 13 3.87(1.18)	0.42^@^	0.07^#^
**Autonomic system**	*n* = 97 5.52(1.44)		*n* = 35 5.26(1.41)		0.36^#^	
	**HIV + **	**HIV-**	**HIV + **	**HIV-**	**HIV + **	**HIV-**
	*n* = 58 5.62(1.53)	*n* = 39 5.37(1.31)	*n* = 22 5.13(1.38)	*n* = 13 5.48(1.49)	0.19^#^	0.81^#^
**Total abnormal reflexes**	*n* = 95 4.34(3.20)		*n* = 35 4.46(2.24)		0.811^@^	
	**HIV + **	**HIV-**	**HIV + **	**HIV-**	**HIV + **	**HIV-**
	*n* = 56 6.04(2.87)	*n* = 39 1.90(1.74)	*n* = 22 5.23(2.02)	*n* = 13 3.15(2.03)	0.17^@^	0.04*^#^

When n values in subgroups are lower than the total group, infants were unable to complete the examination due to fragility or were awake for the habituation section

^#^Equal variance assumed according to Levine’s test for equality of variances

^@^Equal variances not assumed according to Levine’s test for equality of variances

^*^p value significant at the 95% confidence level

## Discussion

This study intended to examine the neurobehavioural functioning of HIV exposed infants, and compare these with the neurobehavioural functioning of HIV unexposed infants born at the same hospital. An interesting outcome was noted in infant anthropometrics, even with LBW as an exclusion criteria. The infants born to the HIV positive mothers, who were all on highly active antiretroviral treatment, currently in the option B + protocol, were smaller than the infants born to HIV negative mothers in all anthropometric measurements recorded. This supports the findings of some studies, indicating that infants exposed to ART have lower birth weight [32, 33].

HIV exposed infants received inferior scores for social interaction, a measure of their ability to orientate to animate and inanimate visual and/or auditory stimuli, a finding synonymous with a previous study demonstrating HIV exposed infants to have inferior orienting and abnormal reflexes [18]. Infants are involved in social interactions from birth, and use these skills in developing a relationship with their parents to elicit the caregiving and nurturing they need [34]. They develop in a social-emotional context, and the dynamic, bidirectional relationship between the infant and child based on the interpretation of this social interaction influences future development [35]. This social interaction, or orientation activities, enhances the connection between the infant and parent by the infant demonstrating their ability to respond purposively to the parent and others in the environment, and things [1]. Suboptimal social interaction may negatively influence the development of this bidirectional relationship. The parent may think that the infant does not want to or cannot interact with them in a meaningful manner and spend less time eliciting this and socially communicating and playing with their infant. This may lead to the infant limiting their development in engaging with the world due to insufficient information being gathered from social interactions, and inadequate interpretation of these [36].

There was a significant difference between the scores achieved by the HIV exposed and non-exposed infants in the motor system. The lower mean score for the general tone in the HIV exposed group indicates that these infants responded to handling with an average tone less than half the time and may have been hypertonic when handled. This hypertonicity in handling is characterized by a stiff, non-cuddly infant who does not mould into the body of the person holding them. This may be interpreted as rejection to a parent and may influence attachment and early development [1, 37]. HIV exposed infants also showed inferior scores in pull to sit, which may result in their parent or caregiver being afraid of handling them for fear of hurting the fragile infant, and also decrease opportunities for increased alertness and social interaction [1]. The lower mean score in the measure of activity level among the HIV exposed infants indicates less spontaneous and elicited motor activity shown by these infants. Motor activity is used by the infant to gain and hold a caregiver's attention, thus ensuring that their physiological and emotional needs are met. A decrease in activity may lead to deprivation of some of these needs, and influence development [34].

State organization is a useful skill, and needed for the infant to cry or fuss to get attention and communicate needs, calm down to feed and interact with caregivers and the environment, and have adequate sleep–wake cycles. Ideally, the infant would transition smoothly between states, and use a variety dependant on their current need. Sleep states are necessary for repair, growth and adequate rest, quiet alert states are necessary for social interaction and learning, and fussing or crying is needed to communicate urgent needs [1]. The HIV exposed infants have impaired functioning in this area, indicating that they are less available to the outside world and thus miss opportunities to shape and enhance their social, emotional, intellectual, language and motor development [1, 37].

Congruent with the study by Scaffidi & Field in 1997, the HIV exposed infants in this study had a higher number of abnormal reflexes [18]. This may be related to later motor delay observed in breastfed HIV exposed infants whose mothers received ART during pregnancy [18, 38]. The higher number of abnormal reflexes noted could be due to the impact of increased stress from exposure to antiretroviral drugs as well as the HIV in utero, with resulting changes in the way the brain functions [39, 40]

The seven supplementary items in the NBAS assessment indicated inferior functioning of the HIV exposed infant. These infants were less available in the quiet alert state needed for learning from interaction with people and the environment. They also had a higher cost of attention in participation in the assessment, becoming more dysregulated and disorganized in their behavioral responses. This level of disorganization will occur during routine handling of the infant, especially in social situations where the infant is passed to and handled by different people. They also required more effort and facilitation from the examiner to transition into a state of arousal where the assessment could be completed. Persistent examiner action and effort is needed in strategies like rocking, containment and allowing the infant to suckle [1]. These HIV exposed infants are more irritable to stimuli and handling, less able to regulate their state of arousal, and less robust in coping with the loading of multisensory stimuli from participation in the assessment. This makes them unavailable for rewarding and meaningful social interaction and thus less responsive to developmental opportunities in the environment [1, 41]. The infant’s ability to regulate their behavioral state in the neonatal period is indicative of the integrity of myelination of the vagal system in the last trimester of pregnancy, which forms a neural base for the development of motor skills and social behavior. The HIV exposed infant, who in this study was exposed to ART for their entire gestation, may have altered development in the vagal system myelination and subsequently altered cortical regulation of arousal [42].

In this cohort, the prevalence of postnatal depression, at 72% was notably higher than previously reported rates in developing countries of 19.8% to 42.4% [43–45], with no statistical significance between the HIV positive and HIV negative group. The reasons for this may be attributed partially to birth type and the physical and emotional difficulty experienced in recovering from birth [46, 47]. “Baby Blues”, with symptoms including tearfulness, fatigue, emotionality, anxiety, and muddled thinking, are a common manifestation in the postpartum period, reported in up to 55% of women globally, usually occurring 1 week to 10 days after birth [48]. A possible reason for a lack of statistical significance between the HIV-positive and HIV-negative groups regarding depression could be that risk factors for developing depression during pregnancy and in the post-partum period include low social support, unemployment, low household income, single status, all factors that are largely experienced by this cohort of mothers making use of this state hospital [49, 50]. A more indepth analysis of the correlations of maternal mental health with perceptions of caregiving competency in this cohort showed no statistically significant correlations between clinical variables (including HIV status and years on ART) and depression but did indicate statistically significant correlations between depression and employment (*p* = 0.01), and birth type (*p* = 0.03) six weeks after giving birth [50]. The infants born to mothers with possible depression performed better in motor skills, state organization, and state regulation than infants born to non-depressed mothers. This is a surprising result, and in contrast with studies, also using self-report measures as an indicator for possible depression, that indicate the poorer performance of these infants in: state organization, state regulation, orientation, alertness, cuddliness, and irritability [21–26]. This may be due to stress reactions in the foetus, with activation of the hypothalamo-pituitary axis and increased cortisol secretion which may result in the infant being more adaptable to external stresses from the changing, dynamic extra-uterine environment as they have more experience in dealing with stress [20].

## Conclusions

HIV exposed infants have notably inferior neurobehavioral functioning when compared to unexposed infants. They experience an altered gestation, with exposure to the HIV virus and to ART taken by their mother in the pregnancy, followed by ART taken orally post-delivery as prophylaxis and via breastmilk as an infant. These infants are medically stable. However, their functioning on a neurobehavioral level, which affects their quality of life and developing relationships with caregivers, is inferior. This may have lasting effects on overall development and may contribute toward some of the developmental, behavioral and mental health difficulties observed in later childhood. Increased vigilance from healthcare providers is needed in the follow-up of these infants. Moreover, guidance for caregivers in assisting these infants in state organization, regulation, motor control and social interaction to buffer the effects of ART exposure and HIV exposure in the period from conception to beyond birth remain essential.

## Limitations

The specific challenges in this study included the administration of the NBAs assessment and maternal consultation in a full obstetric ward with a high turnover rate. Assessments had to be completed by 12 pm daily, as visiting hours for remaining inpatients were from 1 pm, and discharge or transfer to the step-down hospital for others occurred from 12:30 pm. Many confounders were not measured and instead used as exclusion criteria in an attempt to simplify this initial study. There is a need for a larger study where these confounders are considered, as well as the inclusion of the high-risk pregnancy and prematurity. The NBAS is valid in this population as it’s a measure of neonatal neurobehavior, however, only one assessment was reported here. Which provides a snapshot of neurobehavioural functioning, but does not account for change over the neonatal period. Infants who receive sub-optimal scores should be followed up with another NBAS assessment at their well-baby follow-up appointments as a minimum, although it is a lengthy assessment and this may not be feasible in all settings.

## Data Availability

All data generated or analyzed during this study are included in this published article.

## Abbreviations

NBAS: Neonatal behavioural assessment scale
ART: Antiretroviral therapy
HIV: Human Immunodeficiency Virus
EPDS: Edinburugh Postnatal Depression Scale
LBW: Low birth weight
